# Supplementary material for: 3D-visualization of amyloid-β oligomer interactions with lipid membranes by cryo-electron tomography
Source: Chem Sci. 2021 Mar 31;12(20):6896–907. doi: 10.1039/d0sc06426b (PMC8153238; doi:10.1039/d0sc06426b)
Supplement: SC-012-D0SC06426B-s006 [file SC-012-D0SC06426B-s006.pdf]

Electronic Supplementary Material (ESI) for Chemical Science.
This journal is © The Royal Society of Chemistry 2021

Supporting Information

3D-Visualization of Amyloid-β Oligomer and Fibril Interactions with Lipid Membranes by Cryo-Electron Tomography

Yao Tian, Ruina Liang, Amit Kumar,

Piotr Szwedziak* and John H. Viles*

Supplemental Figures: Figures S1- S13

Supplemental Table: Table S1-S2

Movies:

Movie M1: Tomogram of the vesicle shown in Figure 3C. Scale bar: 25 nm.

Movie M2-M4: Single threshold surfaces showing 3D representations of Aβ42 protofibrils shown in the rimmed area of Figure 4A. Scale bar: 10 nm.

Movie M5: Tomogram of the vesicle shown in the top left panel of supplemental Figure S8. Scale bars: 50 nm.

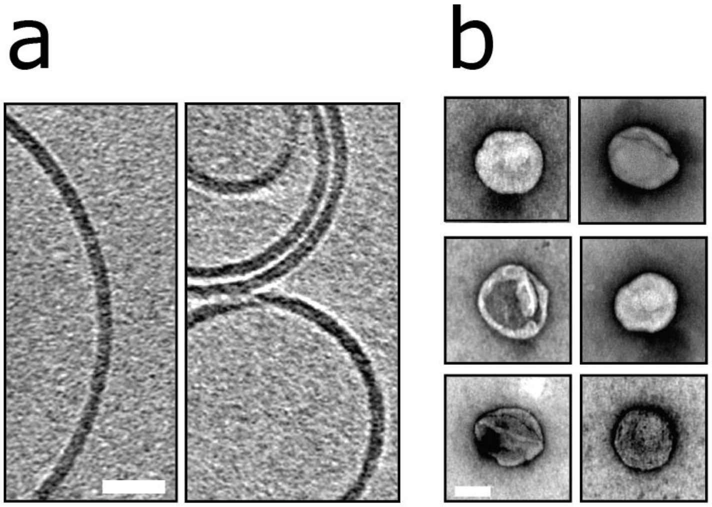

Figure S1: Lipid Vesicles. Liposomes were generated by the extrusion method using a lipid mixture of PC: Cholesterol: GM1, with a lipid of ratio 68: 30: 2 by weight. **a)** CryoET indicates large unilamellar vesicles, typically 100-250 nm in diameter are produced by this method. These are spherical and have a smooth regular appearance where the inner and outer leaflets of the lipid bilayer are indistinguishable by their appearance. There are also examples of multivesicular and occasionally multilamellar liposomes. Tomographic slices are 7.6 nm thick, scale bar: 25 nm. **b)** Vesicles are imaged by negative-stain TEM for the same vesicle preparation, stained with uranyl-acetate. The vesical remain largely circular and intact. These vesicles have a deflated appearance due to the drying effect of negative-stain, scale bar 100 nm.

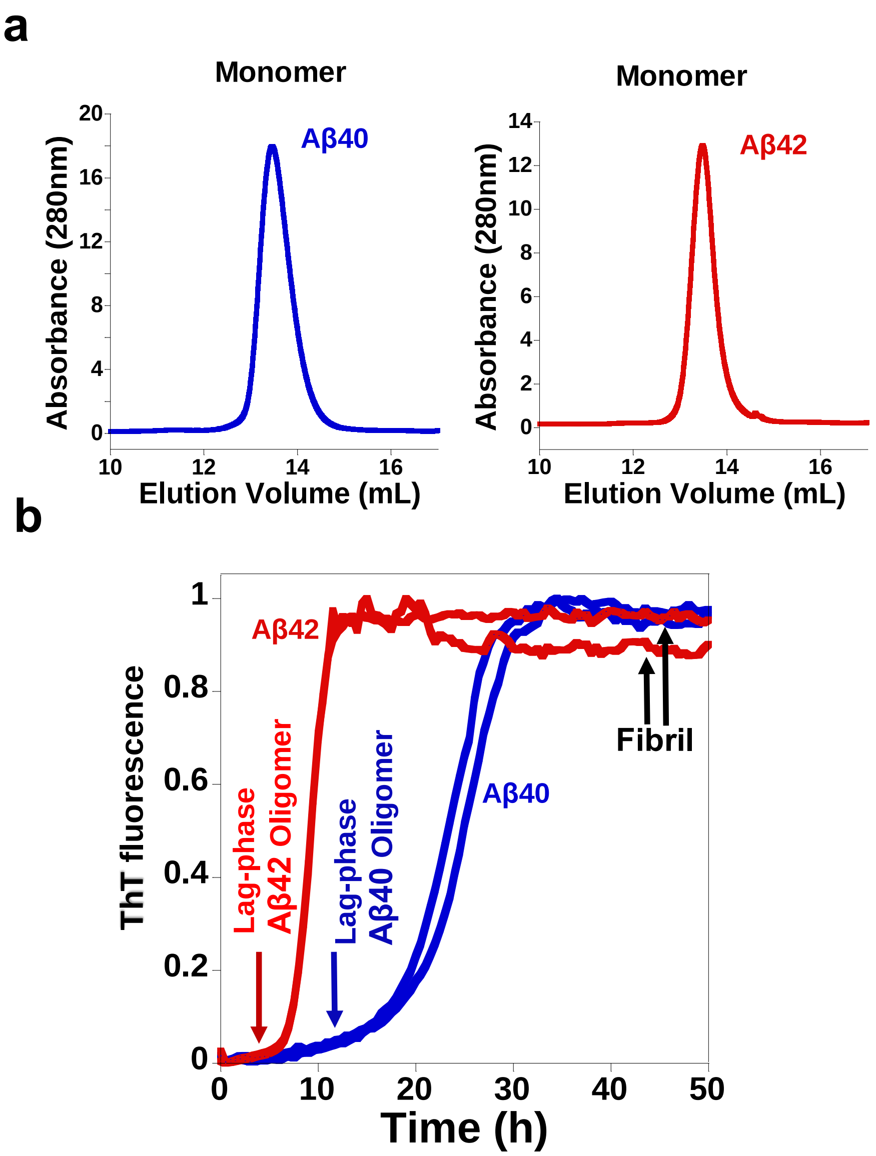

Figure S2: Isolation of Aβ monomer, oligomer and fibril. a) Solubilised recombinant and synthetic Aβ40, and Aβ42 was purified by elution through size-exclusion column (Superdex 75 10/300 GL column). The elution profile (280 nm) indicates a single monomeric fraction of recombinant Aβ40 and Aβ42. The Aβ monomeric samples were taken directly from the SEC column. **b)** Samples designated oligomeric Aβ were taken from wells towards the end of the lag-phase while fibril samples were taken from the well plate once ThT fluorescence signal had plateaued. EM images are shown in Figure S3 and S4. ThT fluorescence fibre growth assays for recombinant Aβ40 and Aβ42 (10 μM) in aqueous buffer containing NaCl (160 mM), HEPES (30 mM), at pH 7.4. Aβ samples (in which ThT had not been added) were taken from the same well-plate at the appropriate time-points.

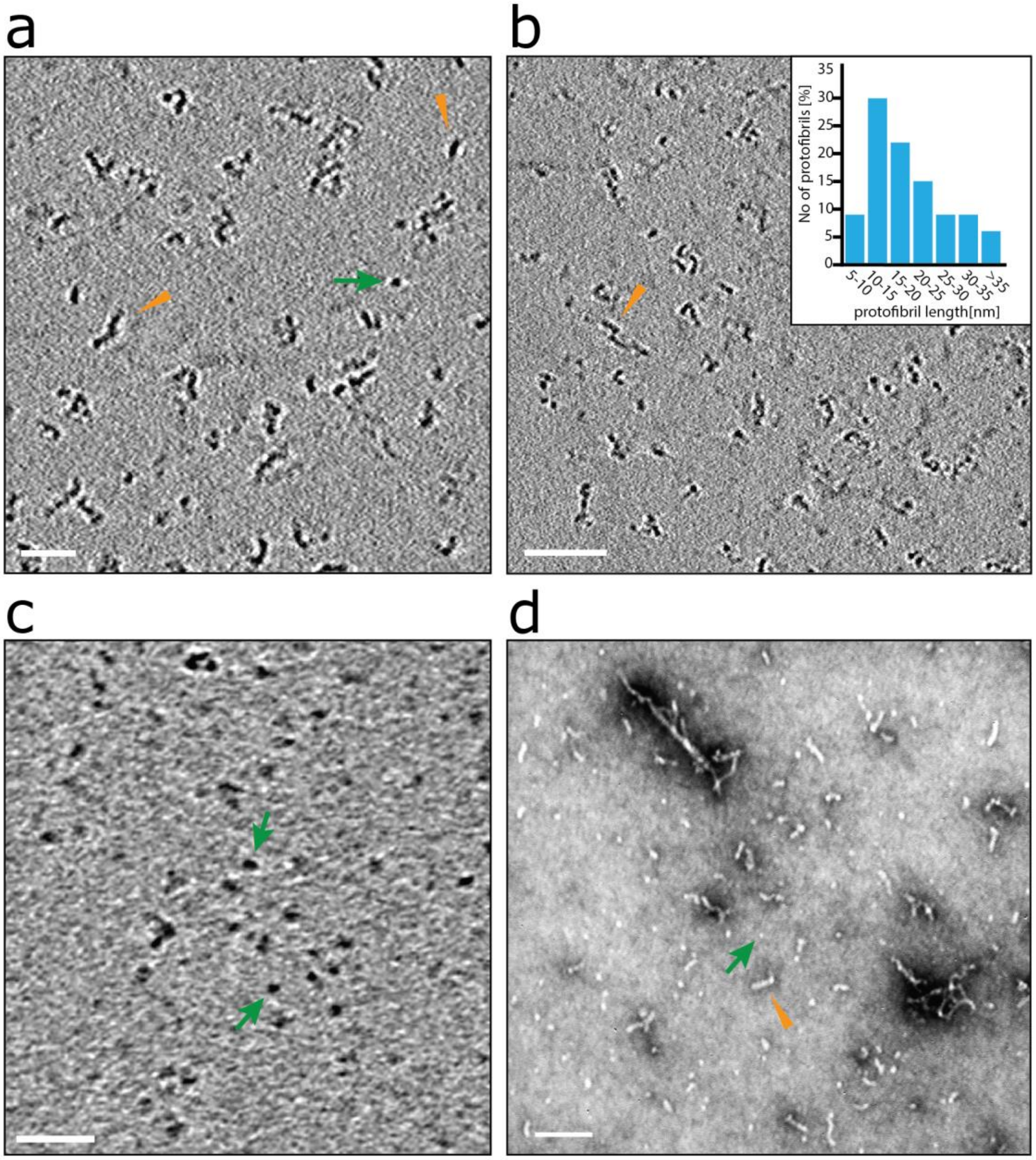

Figure S3: Lag-phase Aβ42 oligomers and curvilinear protofibrils, imaged by cryoET and negative-stain TEM. a) and b) The range of curvilinear protofibrils observed in a tomographic slice (7.6 nm thick) showing Aβ42 protofibrils assemblies (orange arrowheads). The assemblies are very variable in their curvature and can be branched. These structures are typically observed on the carbon support and air/water interface. Histogram shows the range of protofibril lengths (n=100), the majority of curvilinear protofibrils are between 10 and 25 nm, and tend not to exceed 40 nm. Protofibrils diameters are consistently measured to be 2.7±0.4 nm. **c)** Also observed are shorter protofibrils, described as oligomers (green arrows), approximately 3 nm in diameter but can be longer as they become curvilinear protofibrils. The spherical oligomers of *ca* 3 nm diameter suggest a molecular weight of between 12-21 kDa. Using the relationship: Volume (nm^3) = Mass (kDa) * 1.27 (nm^3/kDa). **d)** The same preparations are imaged by negative-stain (uranyl-acetate) showing curvilinear protofibrils and oligomeric structures. Note at 4.2 kDa Aβ monomer and dimers are too small to be visualized by EM. Scale bars: a=25 nm; b=50 nm; c=25 nm; d=50 nm.

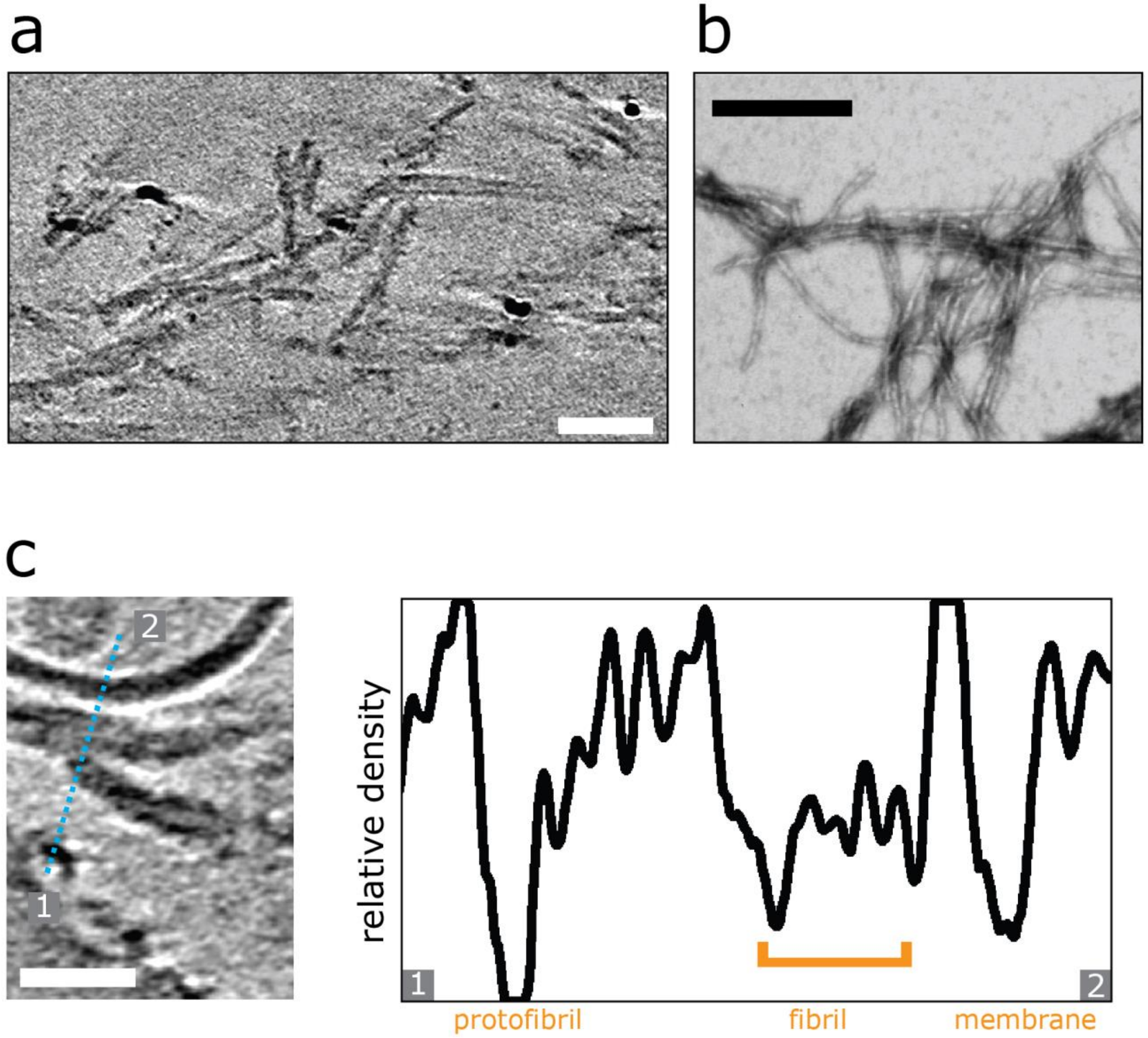

Figure S4: Aβ42 fibrils imaged by cryoET and negative-stain TEM. a) Tomographic slice (7.6 nm thick) for Aβ42 fibrils (10 µM). It is notable the image contrast for fibrils is less than that for the oligomers and curvilinear protofibrils. Scale bar: 50 nm. **b)** TEM negative-stain (uranyl-acetate) image of Aβ42 fibrils. Fibrils are typical 10 nm in diameter and many 100's nm in length, structures are unbranched. Scale bar: 200 nm. **c)** Tomographic slice showing Aβ42 protofibrils and fibrils (left panel) along with the respective density plot (right panel) indicating that the Aβ42 protofibril species have greater density that the fibrils. Scale bar: 25 nm.

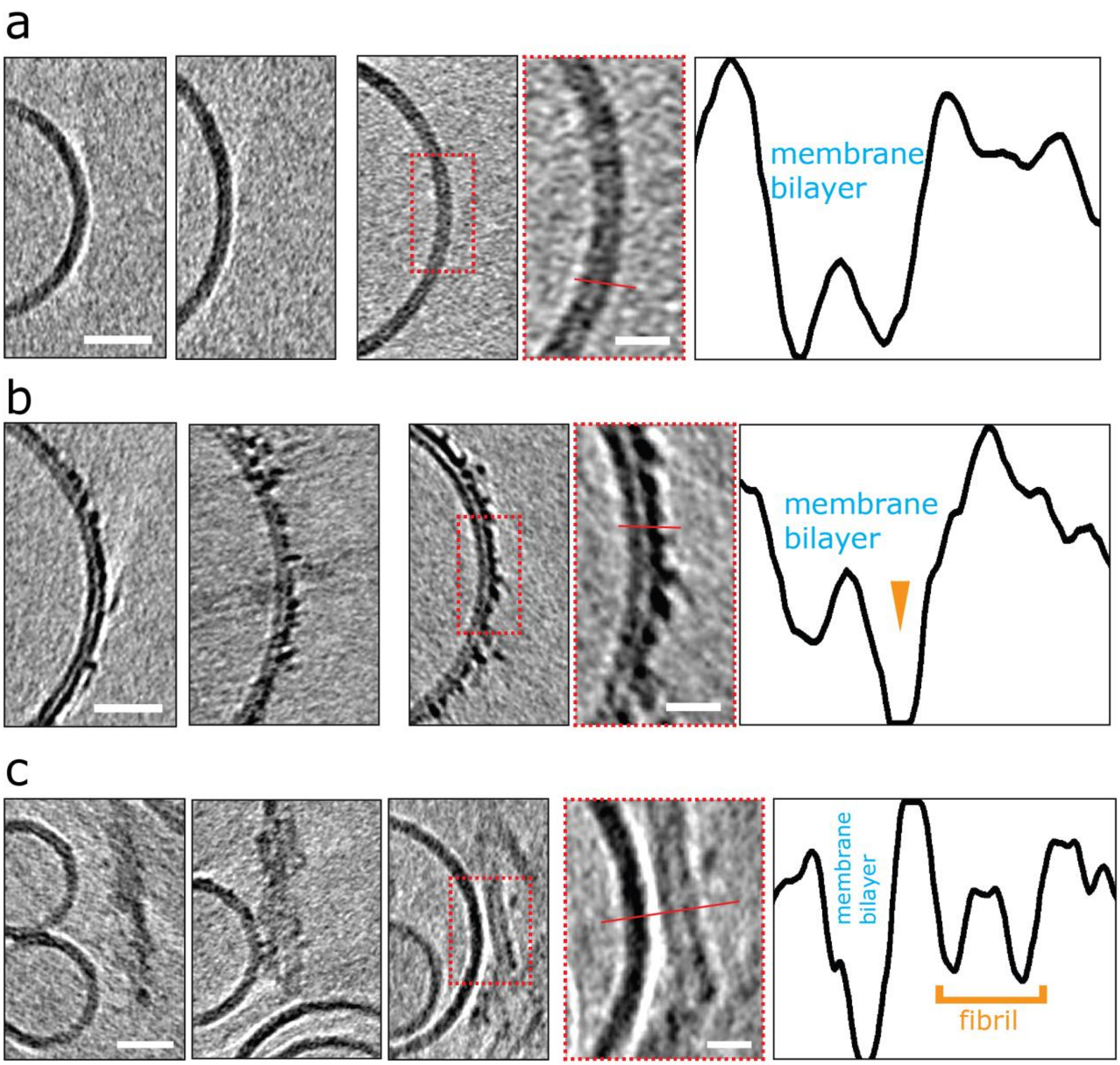
a
membrane
bilayer
b
membrane
bilayer
c
membrane
bilayer
fibril

d

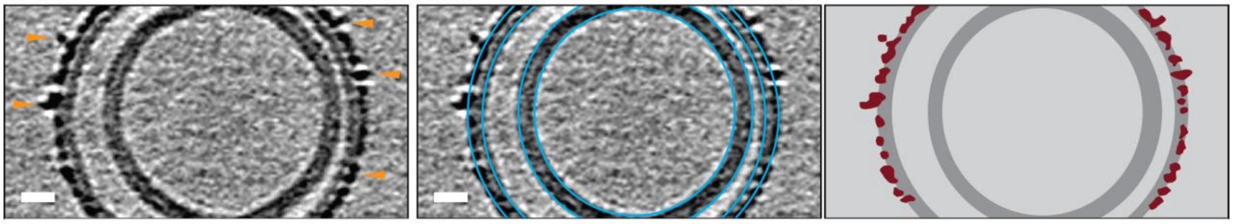

Figure S5: **CryoET images compare the impact of Aβ42 monomers, protofibrils and fibrils on lipid vesicles.** Tomographic slices (7.6 nm thick) showing **a)** monomer **b)** lag-phase oligomers/protofibrils **c)** fibrils. Only Aβ oligomers and curvilinear protofibrils decorate the outer surface of the bilayer. Scale bars: 25 nm. Areas rimmed in red are presented with more detail in the right outermost panels. Scale bars: 10 nm. The insets highlight the increased density on the outer leaflet of the oligomer preparation (b), while for the fibril preparation there is a lack of density even though the lateral face of the fibril aligns closely with the surface of the membrane (c). Profile plots (right panels) along the red lines indicate the asymmetry of the lipid bilayer density in (b) indicating that the outer leaflet is populated with Aβ oligomers and protofibrils (orange arrowhead). The density corresponding to a fibril in (c) is at a distance from the lipid bilayer indicating no membrane association. **d)** A tomographic slice where Aβ oligomers and protofibrils embedded in the lipid bilayer are marked with orange arrowheads (left panel), middle panel represents membranes highlighted with blue circles, and the right panel shows a segmentation with Aβ protofibrils (burgundy) inserting into the lipid bilayer (dark grey). Please note that the inner vesicle is not decorated with Aβ. Scale bar: 10 nm.

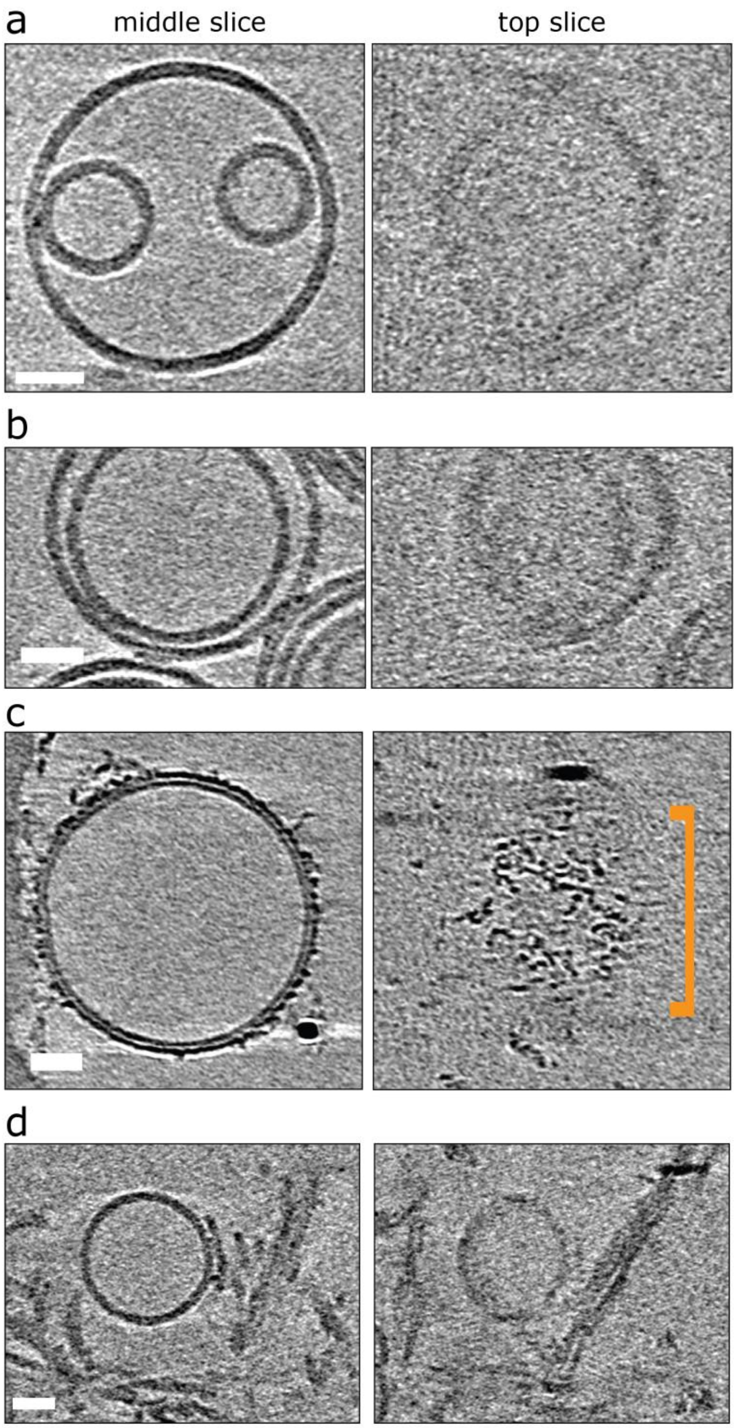

Figure S6: **Top and middle slices of cryoET images compare the impact of Aβ42 monomers, protofibrils and fibrils on lipid vesicles.** Tomographic slices (7.6 nm thick) showing vesicles mixed with various Aβ42 species. Left panels are middle sections, right panels are top sections. **a)** no Aβ **b)** Aβ42 monomer **c)** lag-phase oligomers/protofibrils **d)** fibrils. Please note that only vesicles in panel (c) are decorated with Aβ42 (orange marker). Scale bars: 25 nm.

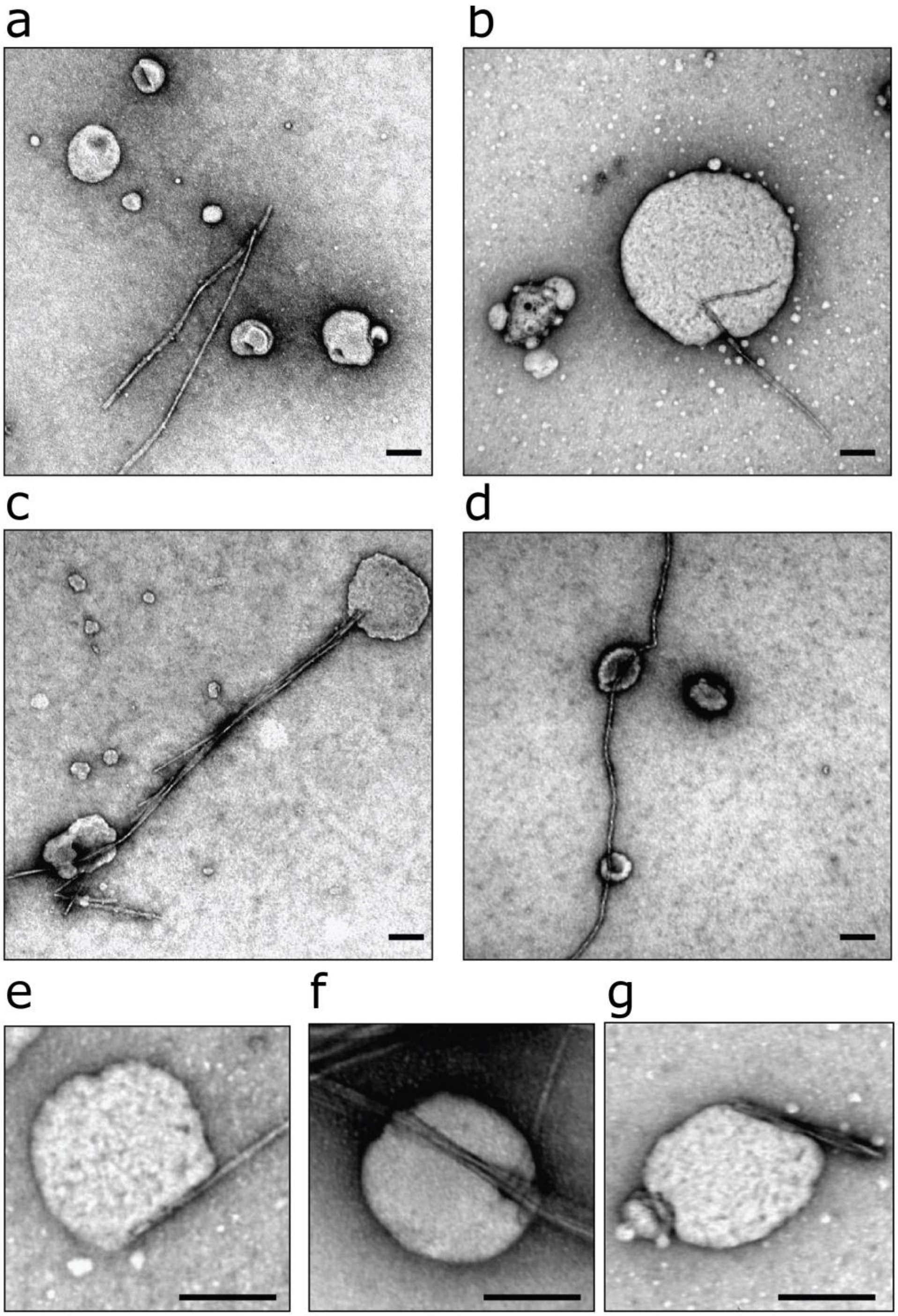

Figure S7: **Negative-stain TEM of vesicles with Ab42 fibrils. a)** Typical negative-stain (uranyl-acetate) image shown; the lateral face of the fibril does not readily adhere to the membrane. There are also some less common examples of fibrils interacting with membrane, these are anchored or restricted to the ends of the fibrils **(b-g)**. Lipid vesicles contain, PC:Cholesterol:GM1 (68:30:2, by weight) 0.05 mg ml^{-1}, pH 7.4. Scale bar: 100 nm.

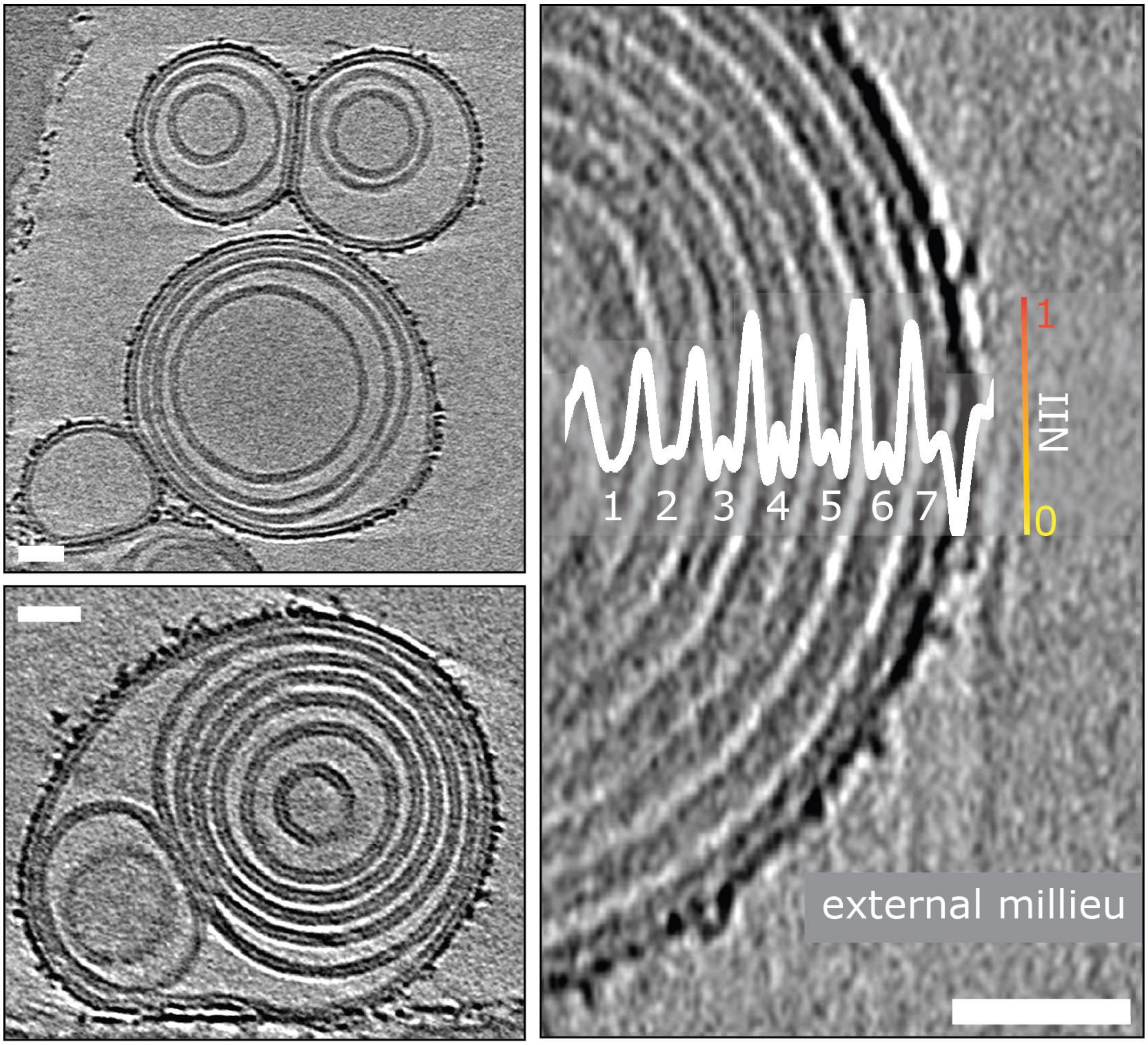

Figure S8: **Aβ oligomers and protofibrils do not migrate to the interior of the liposome.** Tomographic slices (7.6 nm thick) showing that only the outermost leaflet can be decorated with Aβ42 protofibrils whereas the inner layers are protected. Examples of multilamellar vesicles are shown. See Movie M5 for a tomogram of the vesicle shown in the top left panel. On the right panel seven concentric lipid bilayers (numbered) are present. The overlaid NII intensity plot (white) indicates that the outer leaflet of the outermost lipid bilayer (7) is densely packed with Aβ42 oligomers whereas bilayers (1-6) are of similar densities to the inner leaflet of bilayer (7) and devoid of Aβ42. Scale bars: 25 nm.

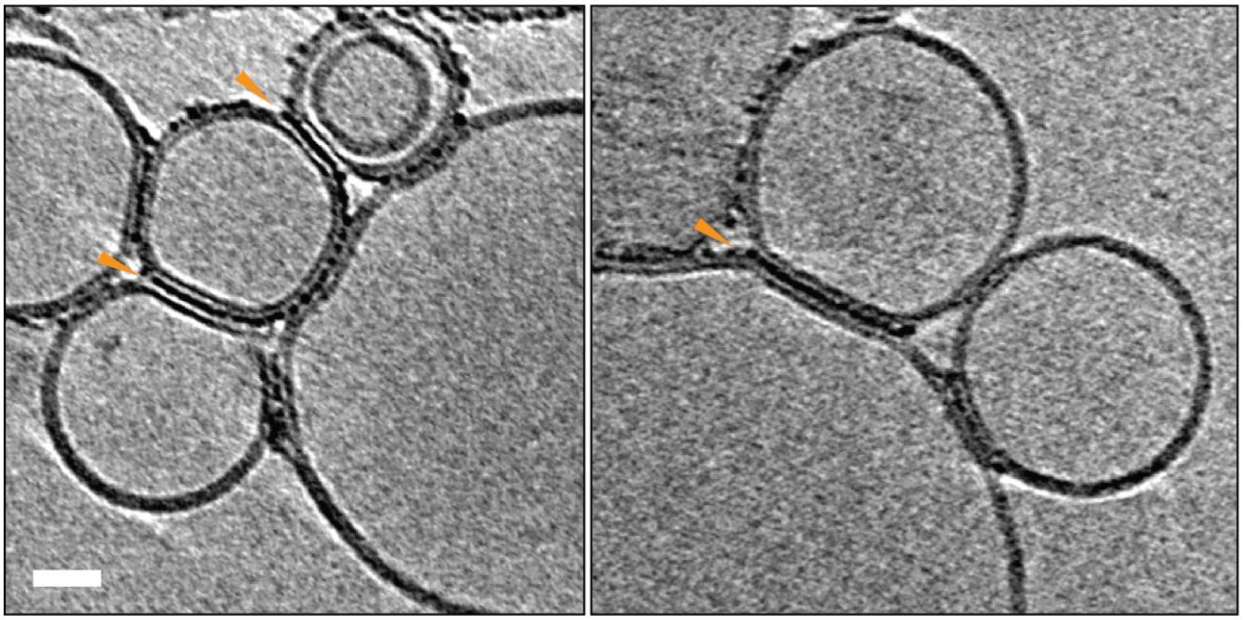

Figure S9: Liposomes can be linked together by Aβ42 protofibrils: For preparation with reduced levels of Aβ42 oligomer/protofibrils, Aβ42 assemblies cluster at the intervesicular space and connect the membranes of neighboring vesicles (orange arrowheads). Aβ42 is not observed elsewhere on the vesicles. The tomographic slices are 7.6 nm thick, with scale bar: 25 nm.

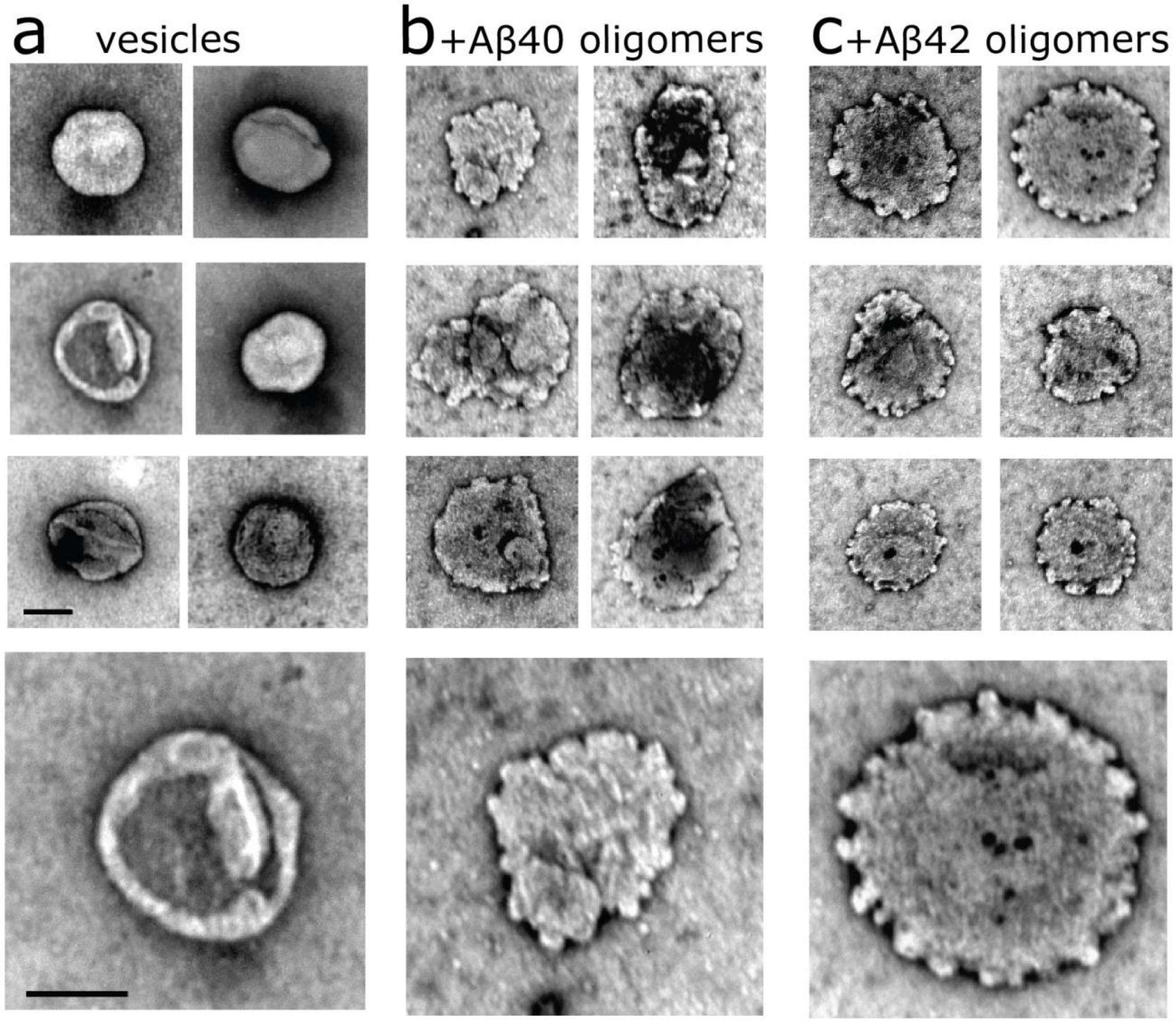

Figure S10: Negative-stain TEM images of vesicles with Aβ oligomers. Aβ40 and Aβ42 oligomers **(b** and **c)** (10 μM) disrupt lipid vesicles. In the presence of uranyl-acetate, negative stain, oligomer cause curvature and budding-off of membrane, while vesicles in the absence of Aβ **(a)** have relatively smooth surface. Bottom row shows enlarged images from columns above. Lipid vesicles contain, PC:Cholesterol:GM1 (68:30:2, by weight) 0.05 mg ml^{-1}, pH 7.4. Scale bar: 100 nm. See quantification of the data in Supplemental Table S1.

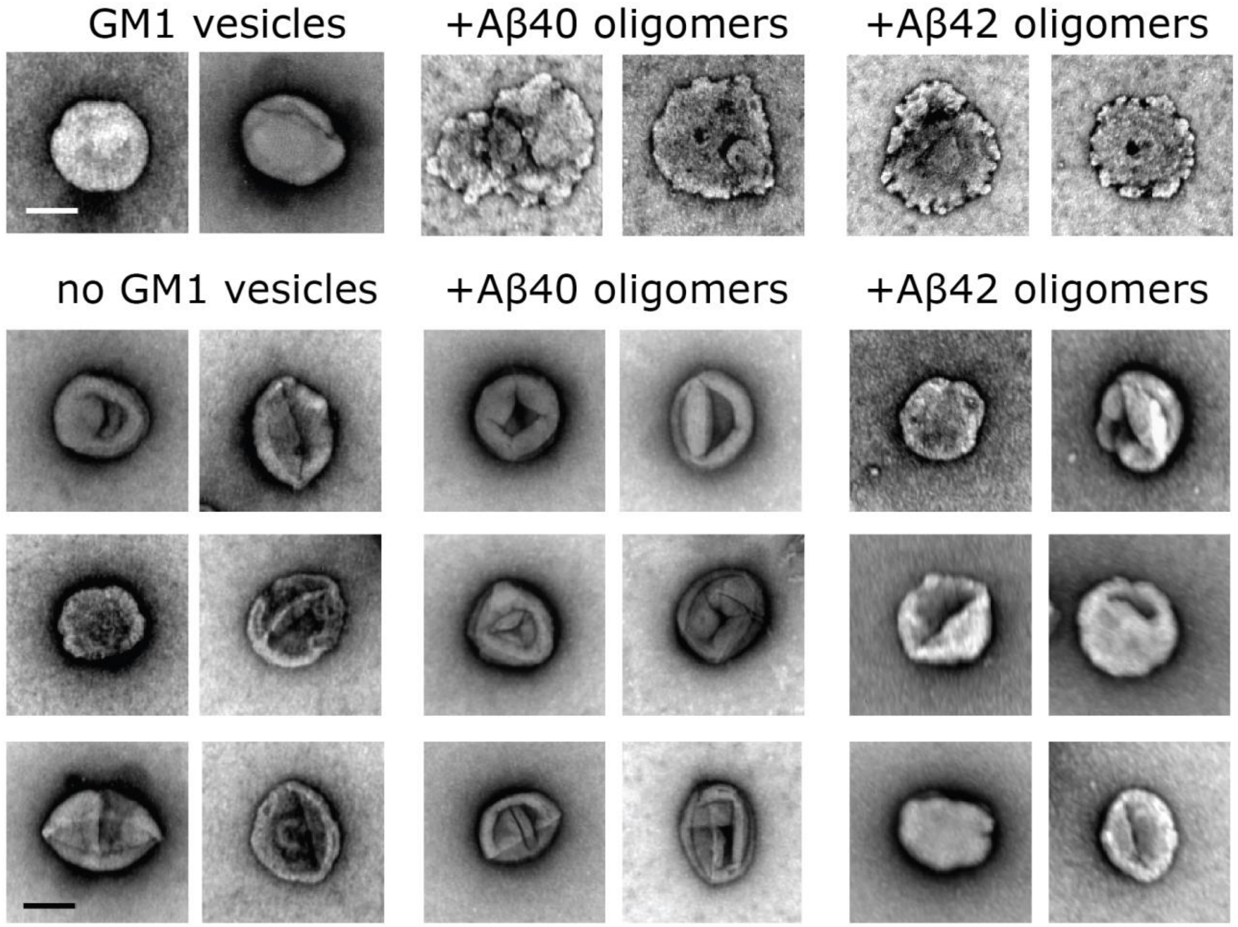

Figure S11: Negative-stain TEM indicates GM1 is important for Aβ-lipid bilayer interactions. Negative stain (uranyl-acetate) TEM of vesicles with Aβ oligomers. Vesicles that do not contain GM1, PC:Cholesterol only (70:30, by weight) are relatively unperturbed by Aβ40 and Aβ42 oligomers. The top row shows control the wide-spread disruption by Aβ for vesicles contain 2% by weight of GM1, as shown in supplemental Figure S10. Vesicles (0.05 mg ml^{-1}) were incubated with Aβ oligomer (10 μM) for 2 hrs at pH 7.4. Scale bar: 100 nm.

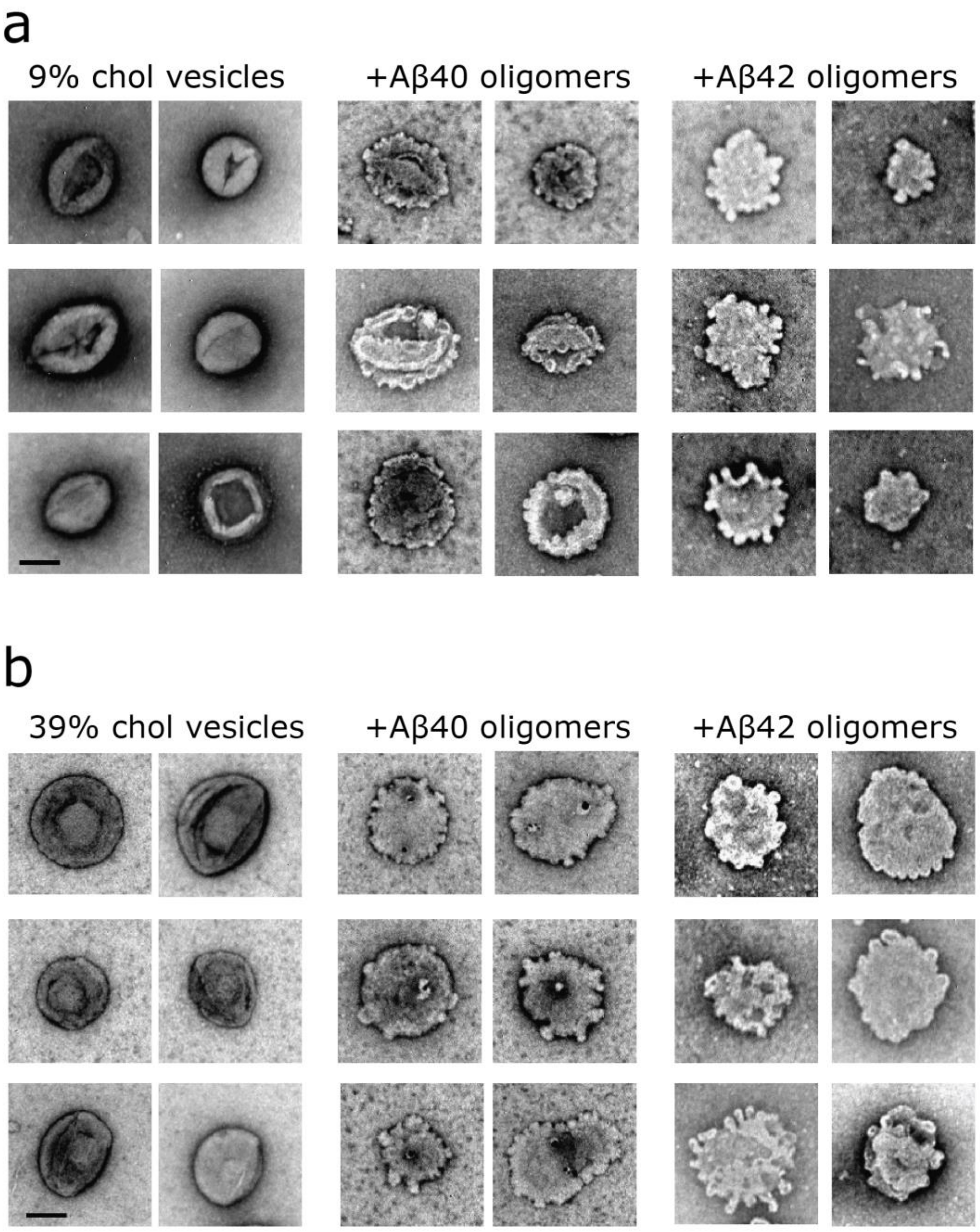

Figure S12: Cholesterol enriched or depleted vesicles do not affect levels of Aβ oligomer induced membrane disruption. Aβ40 and Aβ42 oligomers disrupt lipid vesicles irrespective of cholesterol levels. Negative-stain (uranyl-acetate) TEM of vesicles with and without Aβ oligomers. **a)** depleted cholesterol 9% (with 89% PC by weight). **b)** cholesterol enriched vesicles 39% (with 59% PC). All lipid vesicles contain 2% GM1. Vesicles (0.05 mg ml^{-1}) incubated with Aβ oligomers (10 μM) for 2 hrs at pH 7.4. Scale bar: 100 nm.

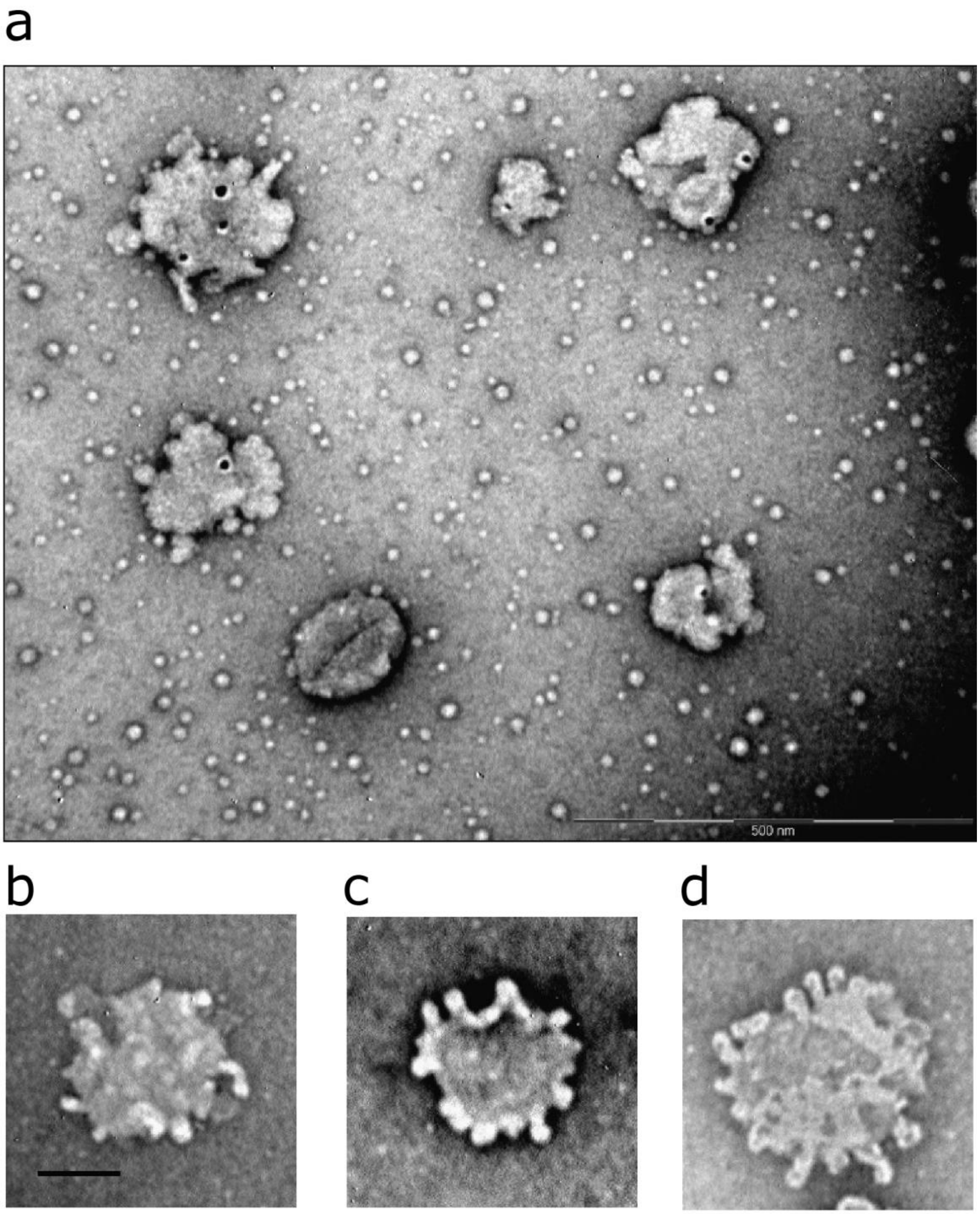

Figure S13: Aβ oligomers induces budding-off of membrane and lipid micelle formation when in the presence of uranyl-acetate. Negative-stain (uranyl-acetate) TEM of vesicles with Aβ oligomers. **a)** Aβ40 on 39% Cholesterol vesicles. **b)** Aβ42 on 9% Cholesterol vesicles. **c)** Aβ42 on 9% Cholesterol vesicles. **d)** Aβ42 on 39% Cholesterol vesicles (B-D are enlarged images from Supplemental Figure S12. All lipid vesicles contain 2% GM1. Vesicles (0.05 mg ml^{-1}) incubated with Aβ oligomers (10 μM) for 2 hrs at pH 7.4. Scale bar: 100 nm.

Supplemental Table S1: Analysis of liposomes decorated by Aβ42 or Aβ40 preparations for negatively stained samples

Liposome Preparation	Number of vesicles inspected	% of vesical decorated
Buffer Only	100	0%
Aβ42 Monomer	100	8%
Aβ40 Monomer	101	9%
Aβ42 Oligomer	100	85%
Aβ40 Oligomer	102	88%
Aβ42 Fibrils	101	15%
Aβ40 Fibrils	100	12%

Typical examples of the vesical images are shown in supplemental Figure S10

Supplemental Table S2: Analysis of liposomes with lack GM1 decorated by Aβ42 or Aβ40 preparations for negatively stained samples

Liposome Preparation No GM1	Number of vesicles inspected	% of vesical decorated
Buffer Only	102	0%
Aβ42 Oligomer	104	16%
Aβ40 Oligomer	100	15%

Typical examples of the vesical images are shown in supplemental Figure S11
