# Supplementary material for: 3D-visualization of amyloid-β oligomer interactions with lipid membranes by cryo-electron tomography
Source: Chem Sci. 2021 Mar 31;12(20):6896–907. doi: 10.1039/d0sc06426b (PMC8153238; doi:10.1039/d0sc06426b)
Supplement: SC-012-D0SC06426B-s007 [file SC-012-D0SC06426B-s007.pdf]

Electronic Supplementary Material (ESI) for Chemical Science.
This journal is © The Royal Society of Chemistry 2021

3D-Visualization of Amyloid-β Oligomer and Fibril Interactions with Lipid Membranes by Cryo-Electron Tomography

Yao Tian, Ruina Liang, Amit Kumar, Piotr Szwedziak* and John H. Viles*

Supplementary Materials and Methods

Aβ recombinant expression

The expression and purification of recombinant Aβ40 and Aβ42 peptide was carried out using the protocol described by Walsh et al [1] . Briefly, the MAβ(40/42)pETSac plasmid of Aβ40 or Aβ42 were transformed into E. coli BL21 (DE3) cells. The protein expression was induced by 1.0 mM isopropyl thio-β-D-galactosidase (IPTG) at an OD600nm of 0.5-0.6. After 4 hrs induction, cell cultures were centrifuge for 15 min at 9000 g at 4 °C, and resuspended in buffer with 10 mM Tris-Cl, 1 mM EDTA at pH 8.5. Cells were then sonicated for 30 s for one cycle at 22% amplitude (4 W). The suspension was centrifuged in 15 min at 25,000 g at 4 °C. The sonication and centrifugation steps were repeated once. The resulting pellet, containing Aβ, was dissolved in 12.5 mL denaturant buffer (8 M urea, 10 mM Tris.Cl, 1 mM EDTA at pH 8.5) with stirring overnight at 4 °C. The suspension was sonicated for 30 s and centrifuge for 15 min at 25,000 g at 4 °C to remove insoluble material. The supernatant which contains solubilized Aβ40 or Aβ42 peptide was retained.

The Aβ protein was purified using DEAE-Sepharose ion exchange resin. The peptide was eluted five steps with the same elution buffer (10 mM Tris.Cl, 1 mM EDTA, 125 mM NaCl and pH 8.5). Eluted protein was dialyzed in 20 mM ammonium bicarbonate buffer and subsequently lyophilization was carried out. The lyophilized Aβ was resolublized in 50 mM Tris.Cl, 7 M guanidine-hydrochloride (Gua-HCl) at pH 8.5 (5 ml). Solubilized peptide was further purified with size exclusion chromatography (SEC) on a Superdex 75 16/600 column (GE Healthcare) using either assay buffer (30 mM HEPES, 160 mM NaCl, pH 7.4) or 20 mM ammonium bicarbonate buffer. Aβ was used directly from the SEC elusion or lyophilized (ammonium bicarbonate buffer). The purity of Aβ was verified using 4-20% gradient SDS-PAGE. Aggregation property was assessed by thioflavin-T assay and negative stain electron microscopy.

Synthetic Aβ Peptides

Synthetic Aβ40 and Aβ42 was purchased from EZBiolab Inc in a lyophilized form. The majority of the data shown is for recombinant Aβ42 while images for Aβ42 fibrils were for both recombinant (Figure S6) but also for synthetic Aβ42 fibrils (Figures 2d and S5c). There was no difference in the appearance of vesicles incubated with Aβ assemblies from a recombinant or a synthetic source.

Monomeric Aβ by Size-Exclusion Chromatography (SEC)

The purified lyophilized Aβ40 and Aβ42 peptides were solubilized at 0.7 mg ml^{-1} in water at pH 10. The solution was then placed on a shaker plate with gently rocked for 2 hours at 4°C and stored at -80°C. Monomeric Aβ was isolated using SEC with a Superdex 75 10/300 GL column (GE Healthcare). The column was pre-equilibrated with 4-(2-hydroxyethyl)-1-piperazine-ethanesulfonic acid (HEPES) (30 mM) and 160 mM NaCl buffer at pH 7.4. The flow rate of 0.5 ml min^{-1} was used. SDS-PAGE was used for analyzing the collected fractions for the presence of the monomeric Aβ. The Aβ40 and Aβ42 concentration were determined using UV absorbance at 280 nm (ε = 1280 M^{-1} cm^{-1}). Monomeric samples were immediately stored after SEC elution at -80 °C. Negative-stain TEM and ThT fluorescence confirmed that SEC-purified Aβ was seed-free. Thioflavin T (ThT) fluorescence showed SEC-purified Aβ had no ThT fluorescence signal and exhibited a clear lag-phase, shown in Supplemental Figure S2.

Fibril growth kinetics for Aβ Oligomer and Fibril Preparations

Aβ40 and Aβ42 monomer (10 µM) were placed in in a 96-well plate in NaCl (160 mM) and HEPES (30 mM) buffer at pH 7.4. Fibril growth kinetics were monitored using a fibril-specific fluorescent dye, thioflavin T (ThT) (20 µM). Experiments were recorded using a Fluostar Omega fluorescent plate reader (BMG Labtech, Aylesbury, UK), with an excitation filter at 440 nm and an emission filter at 490 nm. Fluorescence reading at every 30 minute intervals, following well plates were agitated for 30 seconds. Adjacent sample-wells with no ThT added were used in all experiments.

Vesicle Preparation

Large unilamellar vesicles (LUVs) were produced using an extrusion method described previously [2]. The lipids used were egg phosphatidylcholine (PC) dissolved in chloroform (Avanti Polar Lipids inc.); monosialotetrahexoslganglioside (GM1) dissolved in ethanol: H2O (1:1) (Avanti Polar Lipids inc.); and cholesterol dissolved in chloroform (Sigma-Aldrich Company Ltd.). Most of the studies used a mixture of 68:30:2 by weight of PC: chloroform: GM1. Lipid solutions were placed in a fume hood overnight to allow chloroform evaporation. Lipids film were then re-solubilized in NaCl (160 mM) HEPES (30 mM) buffered at pH 7.4, to a lipid concentration of 1 mg ml^{-1}. LUVs were formed from solubilized lipids using a benchtop mini extruder (Avanti Polar Lipids, Alabama, USA). The lipid solution was passed across a polycarbonate membrane with 100 nm pores 21 times. Vesicles were then stored at 4°C until

use, for up to 2 days and characterized by cryoET, Supplemental Figure S1. Vesicles with elevated or reduced cholesterol were also produced, in particular PC:cholesterol:GM1 in varying ratios (89:9:2 and 59:39:2 by weight). In addition, vesicles were generated in the absence of GM1 (PC: cholesterol, 70:30 by weight). Initial studies confirmed freezing vesicles in aqueous buffer had no apparent effect on the morphology according to negative-stain TEM. Unless otherwise stated all other chemicals were purchased from Sigma-Aldrich.

Cryo electron tomography (cryoET) Image processing

Tomographic reconstructions from tilt series were calculated using RAPTOR [3] and the IMOD tomography reconstruction package, followed by Weighted Back Projection (WBP) or Simultaneous Iterative Reconstruction Technique (SIRT) reconstruction with the TOMO3D package [4, 5]. Currently, a vast majority of cryoET reconstructions are obtained using either of the two algorithms. Briefly, the major advantage of WBP is its computational efficiency and high-resolution signal retention but it suffers from sensitivity to the missing wedge problem and high frequency noise. SIRT, on the other side, is computationally expensive but offers visually pleasing, high contrast reconstructions that are easier to interpret than tomograms reconstructed with WBP. Most of the cryoET images are generated with SIRT while images in Fig 3 and S5 are processed using WBP.

WBP is a Fourier-space algorithm. It relies on taking a Fourier transform of each tilt series image, placing these slices in a Fourier space at the appropriate, refined angles and inverse Fourier transform to back project the volume into real-space to arrive at a tomographic volume. Importantly, Fourier components are weighted to prevent oversampling of low-resolution features, which is necessary to properly represent the high frequency information. SIRT is an iterative real-space method. Essentially, it minimizes the error between the experimental data and the equivalent projections derived from the reconstructed volume. The SIRT algorithm in every iteration calculates projections from the current volume, estimates the error between the experimental projections and the computed projections and refines the volume by back-projection of the average error.

Measurements of distances between structures were carried out within IMOD, the length of protofibrils followed the curvature of the protofibrils in three dimensions manually plotting through the center of mass using the 3dmod model modality. Single threshold surface representations and movies were prepared using Chimera [6], using a similar threshold level to exclude most background noise for each data set. The choice of the threshold level is subjective and depends on the density distribution and background artifacts.

Analysis presented in Figure 2, grey-values were measure all around perimeters of vesicles by performing radial averaging using the extended Radial Profile plugin of ImageJ [7]. Profile plots of normalized integrated intensities around concentric circles as a function of distance from a point in the center of each 2D projection of a vesicle were generated using ImageJ.

Negative-stain TEM

Vesicles (0.05 mg ml^{-1}) were imaged by neg-stain TEM in the absence and presence of Aβ (10 µM monomer-equivalent) incubated for 120 mins. 5 µl aliquots of sample were added to glow discharged carbon-coated 300-mesh grids (Agar Scientific Ltd) by the droplet method, then blotted after 90 seconds and rinsed with ddH20. Following this, (5 µl) uranyl acetate (2% g/100 ml) was added, then blotted and rinsed after 10 seconds. Vesicle samples were also stained with phosphotungstic acid (PTA) (2% g/100ml) data not shown. Images were at 80,000 magnification by a JEOL model JEM-1230 electron microscope

(JEOL, Ltd., Japan), operated at 80 kV, paired with a 2k Morada CCD camera and corresponding iTEM software (Olympus Europa, UK).

References

1. D. M. Walsh, E. Thulin, A. M. Minogue, N. Gustavsson, E. Pang, D. B. Teplow and S. Linse, *Febs Journal*, 2009, **276**, 1266-1281.
2. C. J. Matheou, N. D. Younan and J. H. Viles, *Biochem J*, 2015, **466**, 233-242.
3. F. Amat, F. Moussavi, L. R. Comolli, G. Elidan, K. H. Downing and M. Horowitz, *J Struct Biol*, 2008, **161**, 260-275.
4. J. I. Agulleiro and J. J. Fernandez, *Bioinformatics*, 2011, **27**, 582-583.
5. J. R. M. Kremer, D.N.; McIntosh, J.R., *Journal of Structtural Biology*, 1995, **116**, 6.
6. E. F. Pettersen, T. D. Goddard, C. C. Huang, G. S. Couch, D. M. Greenblatt, E. C. Meng and T. E. Ferrin, *J Comput Chem*, 2004, **25**, 1605-1612.
7. C. A. Schneider, W. S. Rasband and K. W. Eliceiri, *Nat Methods*, 2012, **9**, 671-675.
